# Consumption—Its Relation to the General Public

**Published:** 1902-03

**Authors:** J. Lawton Hiers

**Affiliations:** Savannah, Ga.


ATLANTA

### Journal-Record of Medicine.

**Successor to Atlanta Medical and Surgical Journal, Established 1855, and Southern Medical Record, Established 1870.**

Vol. III. MARCH, 1902. No. 12.

BERNARD WOLFF, M.D., EDITOR, Nos. 319-20 Prudential.

Published Monthly.

M. B. HUTCHINS, M.D., BUSINESS MANAGER, No. 64 Marietta St.

### *ORIGINAL COMMUNICATIONS.*

### CONSUMPTION—ITS RELATION TO THE GENERAL PUBLIC.

By J. LAWTON HIERS, M.D.

Savannah, Ga.

Modern medicine is the child of investigation; empiricism the relic of ignorance. The one bids fair to live and grow in wisdom for ages yet unborn; the other to die in ignorance and disrepute. Science and truth beget sympathy; skepticism and superstition indifference and, consequently, stagnation. While, therefore, we recall the darkness of the past with no little pride, for it has made possible the present and given to humanity many of her noblest sons, we see in the future the dawn of an era when preventive medicine shall supplant the treatments now in vogue and beam in gratitude upon a healthful and healthy posterity.

The time is ripe for the cooperation of the laity with the medical profession for the preservation and prolongation of human life; and I welcome the inauguration of a movement which, in the liberality of its scope, is fostered by men in whom "one touch of nature" has made "the world akin." There is no truer aphorism than that "in union there is strength." Standing alone we have accomplished much; working collectively we can achieve more. Enlightenment is the time-piece of progress; and as in retrospection we recall that period when turmoil and strife, war and barbarism joined hands; when ignorance and desolation marched side by side while sorrow and despair followed in their wake, it is a pleasant thought that the dark age is gone. In striking contrast is a day of modern progressiveness and investigation in which man seems to have attained the zenith of his glory and, half inclined to doubt his handicraft, views in amazement the wonderful possibilities of his mind.

Nothing more effectually dampens a wholesome reform than the propagation of abstruse theories or the advocation of ridiculous and unreasonable propositions. One fact is of greater import than a host of conjectures; and to be consistent, must be limited in the lesson it would enjoin on the public.

In June, 1899, at its Columbus meeting, the American Medical Association appointed a committee which was "instructed to report on the general subject of tuberculosis." This report treated at length of its nature, communicability, prevention, its control and the education of the public so as to "lessen their chances of infection, and their prospect of recovery when infected;" and the expediency of establishing national and State sanatoria for the segregation of those who, unfortunately, had fallen victims to its insidious approach. To attempt an elaboration of the deductions formed by the gentlemen composing this committee would be "wasteful and ridiculous excess;" so, appreciating the difficulty of adding to or subtracting from its completeness, it is my purpose to base my remarks chiefly on their conclusions.

Tuberculosis is an infective disease, attacking many tissues of the body, and caused by the growth and development therein of the tubercle bacillus. The lesions formed are characterized by nodular bodies denominated tubercles, which in the course of time may undergo cheesy degeneration, sclerosis, ulceration, or, in some conditions, calcification (Osler). Following its invasion a poisoning of the system results; and unless early arrested eventuates in the degeneration of the tissues or organs involved, the destruction of function, and, in a large proportion of cases, tedious and ultimate death. As the bacilli may be present in the respiratory tract without producing the disease, it follows that the lung tissue must be fertile and conducive to their growth. To employ an agricultural phrase, rough, stony ground will soon choke out their existence, while a rich, prolific soil makes an abundant harvest. Any circumstances, therefore, which tends to lower the tone of the general system renders the tissues more susceptible to their encroachment.

To obtain accurate statistics concerning the mortality of consumption in the United States is difficult,—partly because of the almost impossible task of counting those who yearly succumb to its ravages, and in view of the fact that numerous cases are wrongly diagnosed. However, enough is known to warrant the assumption that from 12 to 14 per cent. of all deaths annually are attributable to this cause. Or, to quote from the report of the committee above alluded to—"Tuberculosis kills thirty times as many as die from smallpox and scarlet fever together, and four and a half times as many as from these two diseases with typhoid fever and diphtheria combined." The foregoing figures but verify the contention so often made, that this is the most pernicious malady that afflicts mankind; and, as has been said, it is, moreover, "the one least subject to fluctuation; the most constant from year to year."

That consumption is a communicable disease is now an established fact. It may be transmitted from one person to another; and depends for its existence upon the susceptibility of the victim attacked. Not every one exposed to its invasion contracts tuberculosis, for if the lung tissue or organ invaded possess a high grade power of resistance no injury results. All of us are more or less subject to its influence, but only those succumb who possess some individual characteristic essential to its life. The air we breathe, the food we eat, the milk we drink frequently contains bacilli in abundance, yet it does not necessarily follow that we suffer thereby, for a perfect physiological function, bodily vigor, exercise and a regard for the laws of hygiene render innocuous the damage that under opposite environments would accrue. It is not this individual who suffers and in whose behalf we plead, but that unfortunate whom nature has not so endowed, and who, on account ill-health, over-work, starvation, and unsanitary surroundings, falls into its ravenous grasp.

As to prevention, our duty then is obvious. To anticipate a complete suppression or eradication is virtually to "hope against hope." Much, however, can be accomplished looking towards its control; but success in a great measure is commensurate with the unanimity of judgment of the medical profession, together with the active co-operation of those whose interest in their fellow man may induce them to lend effective and valuable assistance. The first essential to the limitation of the power of consumption is the diminution of the number of tubercle bacilli in the air we breathe. Looking to the accomplishment of this end I can suggest nothing further than the concise but pertinent facts adduced by the able committee, who, as the representatives of the American Medical Association, have been instructed to present their report "to the Congress of the United States and to the legislatures of the various States of the Union, and urge upon them that appropriate measures be speedily taken." The disease being largely air-borne, they have advocated these radical measures: (a) The destruction as far as possible of all tubercular sputum, either by fire or by chemical agents in spit receptacles; (b) the careful disinfection of contaminated clothing, utensils and rooms—clothing and utensils by heat, either dry or moist, and rooms by formalin; (c) careful management of the patient in his person, and especially as to his expectoration; (d) the segregation of infected persons, in whom the disease is active, under the care of nurses competent to prevent them from contaminating the atmosphere; (e) the protection of food stuffs from pollution, particularly meats and milk, and (f) in those known to have a predisposition to consumption, the careful and constant keeping of the body in as perfect physical condition as possible. This becomes a potent factor when the liability of all classes to infection is considered, and consequently any deviation from this rule may in course of events prove of incalculable harm. It is, therefore, through the instrumentality of fresh air, sunshine and exercise, and these alone, that the individual can escape the doom which hangs in all of its awfulness over him.

In attempting to educate the people to a proper appreciation of the means at hand for preventing the spread of the "great white plague," and in our eagerness to counsel the infected how they may have greater hope of convalescence and ultimate recovery, we should advocate the most common-sense and practical measures. Theories that cannot be satisfactorily demonstrated and glittering technical phraseology are but meaningless platitudes calculated to increase rather than lessen their skepticism, and consequently to thwart any purpose, however good, looking to their betterment. The first element to success, therefore, is that we should descend to a plane level with them rather than endeavor to lift them to a sphere where confusion and a multiplication of incongruous ideas would result. And, again, simplicity, and not abstruseness nor a succession of hardships, should characterize every suggestion made; or, as one writer has tersely remarked: "A few cardinal doctrines ground into public thought will be more efficient than a hundred casual details."

The public should first of all be instructed as to the nature and distribution of consumption. This can be accomplished in numerous ways, any one of which is obvious to an intelligent observer. Posterity might profit did the schools of the State manifest a deeper interest in this and other medical subjects—as personal hygiene and sex matters—that, if mentioned at all, are given but a passing notice. If possible or convenient, those undeniably affected should be separated from the well and isolated at some agreeable retreat or sanatorium. Provided this be out of the question, and they continue to live with their families, they should pay the strictest attention to personal hygiene and conform each act to the laws of health. To advise a tuberculous patient of his condition is, as a rule, to gain his active cooperation in the line of work mapped out for him to follow. To keep him in ignorance is to endanger not only those with whom he is thrown in contact, but the community as well.

On account of our proclivity for spitting, Charles Dickens concluded that the Americans, as a people, lacked the national instinct of cleanliness. The promiscuous expectorating in public places, on the sidewalks, in street cars or other conveyances, should not only be discountenanced but forbidden by legal enactment. Aside from the element of filth connected therewith, the danger accruing to innocent citizens from such a nefarious practice is at once apparent. The sputum should be received in articles that can be readily burned or easily disinfected. Since having a clearer conception of the infectiousness of tuberculosis, and especially cognizant of the danger emanating from the too free license accorded expectorators, several of our cities have passed ordinances prohibiting the indulgence of this act in or about public places, and making an offense punishable by fine or imprisonment, on conviction. Others through the medium of a "general nuisance" statute, can reach offenders on the logical ground that indiscriminate spitting is unsanitary, and, therefore, detrimental to the community. In these latter the respective boards of health are given authority to abate or curtail anything calculated to prove disastrous to their constituents. In proportion as public opinion is being educated, in the same ratio are we overthrowing filth for cleanliness. It is but necessary to have placed on our bureaus of health men of unimpeachable integrity—men known to have the health and welfare of those over whose destinies they preside at heart, not low-born nor corrupt politicians who would shower patronage according to the dollars received—and many of those men doomed to an untimely end would live to bless their homes, their country and their God.

The most scrupulous care is required of the consumptive. He must be carefully managed to prevent the transmission of his malady. Those serving in the capacity of nurses cannot exercise too many precautions, for contamination of the patient's apparel or bedclothing is often a dominant factor in the propagation of other cases. For similar reasons the phthisical should not wear a beard; and carpets, rugs, or anything apt to increase the liability of the infection of others should be removed from his chamber. In this connection it appears eminently proper to suggest that, as in other contagious or infectious diseases, the board of health, or health-officer, as the case may be, should, after death, subject his apartments to a thorough disinfection. A possible exception may be made when possessed of absolute knowledge that the most perfect care and cleanliness were maintained throughout the entire sickness.

Thus far medicines have proven of little efficacy in eradicating consumption or arresting its progress. Judging from the past the future, in this respect at least, warrants little hope. It behooves us, therefore, to instruct the public as to its duty when an unfortunate has been seized with this evil. The principal object to be attained is to "increase the resisting power of the body to the disease"; the substitution, as it were, of cat for guinea-pig tissue. Conservative therapeutics may prove a valuable ally; but, primarily, a radical change in the patient's environments is indicated. If possible an out-door life should be followed, with an abundance of sunlight and fresh air; if this be impracticable, then nature should be simulated in so far as permissible indoors. Fresh, pure air and sunshine, together with a climate free from moisture, are three essentials. Given these elements, and, provided the progress of the disease is not too great, we are reasonably sure of success. Necessary accompaniments are a change of environments and conditions, a reduction in the amount of labor done, an abundance of rest and sleep, and a regulation of the diet to suit the most delicate or susceptible stomach—taken, of course, with greater frequency than in the normal state.

Another circumstance of importance in combating this disease is the closest scrutiny over the preparation of the various food stuffs, and the inspection of dairy herds and the destruction of cattle known to be exposed to or infected by the tubercle bacilli. Of such moment is this latter suggestion that, if necessary, the State should see to its enforcement.

The successes held out by the sanatorium treatment are so numerous that the most skeptical are becoming its advocates. The plan so common in Europe is now to a great extent practiced in this country. Patients may, under the watchful eye of a competent nurse, appreciably improve at home; but at most, invariably some blunder will offset the gain accruing therefrom. Within the recesses of such an institution where fresh air is abundant and an outdoor life exacted, the diet regulated and the elements of hygiene followed with clock-like precision, the hope of ultimate recovery, it must be admitted, is enormously enhanced. Statistics prove that the maximum of cases of incipient origin fully recover at these retreats, a large per cent. of those who have passed into the second stage are permanently bettered, and not a few in whom the disease had made greater progress show an improvement. These deductions but verify the contention that in a properly managed sanatorium the average case has a better chance of recovery.

The greatest argument against the sanatorium is its expense. With few exceptions they are institutions in which the patient of average means can little afford to indulge. That they are rapidly on the increase is commendable; but, likewise, are we impressed with the inability of many to patronize them. The poor are as deserving as the rich, and should be accorded equal privileges with them. Their infirmities are just as fatal, their aspirations just as soulful, their lives just as valuable. The State owes them the same prerogative that it affords its other unfortunates. The consumptive, shrunken, emaciated, with ghastly features and a face from which health's crimson blushes have fled; with hollow eyes that have lost their lustre, a hacking cough and a slender frame from which life's blood is slowly ebbing, demands the same care and tender sympathy. The State, therefore, should erect and maintain for them homes where they can receive every necessary attention, and profit in like proportion as do their opulent and oftentimes less deserving companions in sickness. "Health is wealth"; and by thus circumscribing the power of consumption the Commonwealth must of necessity reap a richer reward and hand down to posterity a heritage more lasting than moulded bronze or sculptural adamant.

Now, in conclusion, to summarize :

1. It is the duty of every national, State and municipal government to educate the people along the following lines : (a) consumption is contagious ; (b) it is eradicable.

2. Every citizen infected with tuberculosis should enjoy the prerogative of residing in a tuberculosis hospital; and advised of the benefit accruing therefrom.

3. Sanatoria for the consumptives are as necessary as other State homes or asylums.

4. Even a temporary sojourn in such an institution is benficial, in that it teaches above all things the proper care of self.

5. Fresh air, sunlight and an out-door life form a trinity that cannot be disregarded in bringing about a successful issue.

6. Bad ventilation, confinement, bad drainage and a contempt for hygiene, individually or collectively, assist in the propagation of the disease.

7. Segregation of the infected is essential to the welfare of a community.

8. State, national or municipal laws are necessary to regulate indiscriminate expectoration.

9. In the cabinet of the President there should be a "Secretary of Hygiene," under whose administration and admonition the ultimate control and eradication of this and other scourges of mankind can be effectually realized.

The work is well begun. The pessimist of yesterday is the optimist of to-day. "Preventive medicine is the legitimate work of the State; remedial medicine, except in so far as it may be preventive, is not." It, therefore, devolves on the State as the "custodian of the material prosperity of its people," to awake from its lethargy and follow where science, truth and health may direct.

